# Supplementary material for: HIVprotI: an integrated web based platform for prediction and design of HIV proteins inhibitors
Source: J Cheminform. 2018 Mar 9;10:12. doi: 10.1186/s13321-018-0266-y (PMC5845081; doi:10.1186/s13321-018-0266-y)
Supplement: Supplementary file 2 — Additional file 2. Source code of HIVProtI web server. [file 13321_2018_266_MOESM2_ESM.zip › HIVprotI_Source-code/hivprotI/jsmol/jmol-flot-energy.htm]

|  |  |
| --- | --- |
| animation first previous on off next last |  |
| Drag and drop any Spartan file into the above applet. | Click on a point, or scan along the baseline to move rapidly among the models. |
| This page and jmol-flot.htm illustrate how the Flot graphing package can be used with Jmol to depict molecular data graphically. Note that this page uses the signed applet, so you can drag-and-drop a file from your local file system onto the Jmol applet. The Flot package is quite flexible and very easy to work with. For these pages I've adapted it a bit -- the modified flot JavaScript file is in the flot subdirectory. Mostly I added a hover delay option and the option to hover over a vertical or horizontal slice rather than exactly on a point. For the Ramachandran Plot at jmol-flot.htm, I added the option to have major tick marks drawn in a darker color. | |
